# TSWIFT: Tower Spectrometer on Wheels for Investigating Frequent Timeseries for high-throughput phenotyping of vegetation physiology

**DOI:** 10.1186/s13007-023-01001-5

**Published:** 2023-03-28

**Authors:** Christopher Y. S. Wong, Taylor Jones, Devin P. McHugh, Matthew E. Gilbert, Paul Gepts, Antonia Palkovic, Thomas N. Buckley, Troy S. Magney

**Affiliations:** 1 Department of Plant Sciences, University of California, Davis, Davis, CA 95616 USA; 2 Department of Earth & Environment, Boston University, Boston, MA 02215 USA

**Keywords:** High-throughput phenotyping, Hyperspectral reflectance, NDVI, PRI, Remote sensing, SIF, Vegetation indices

## Abstract

**Background:**

Remote sensing instruments enable high-throughput phenotyping of plant traits and stress resilience across scale. Spatial (handheld devices, towers, drones, airborne, and satellites) and temporal (continuous or intermittent) tradeoffs can enable or constrain plant science applications. Here, we describe the technical details of TSWIFT (Tower Spectrometer on Wheels for Investigating Frequent Timeseries), a mobile tower-based hyperspectral remote sensing system for continuous monitoring of spectral reflectance across visible-near infrared regions with the capacity to resolve solar-induced fluorescence (SIF).

**Results:**

We demonstrate potential applications for monitoring short-term (diurnal) and long-term (seasonal) variation of vegetation for high-throughput phenotyping applications. We deployed TSWIFT in a field experiment of 300 common bean genotypes in two treatments: control (irrigated) and drought (terminal drought). We evaluated the normalized difference vegetation index (NDVI), photochemical reflectance index (PRI), and SIF, as well as the coefficient of variation (CV) across the visible-near infrared spectral range (400 to 900 nm). NDVI tracked structural variation early in the growing season, following initial plant growth and development. PRI and SIF were more dynamic, exhibiting variation diurnally and seasonally, enabling quantification of genotypic variation in physiological response to drought conditions. Beyond vegetation indices, CV of hyperspectral reflectance showed the most variability across genotypes, treatment, and time in the visible and red-edge spectral regions.

**Conclusions:**

TSWIFT enables continuous and automated monitoring of hyperspectral reflectance for assessing variation in plant structure and function at high spatial and temporal resolutions for high-throughput phenotyping. Mobile, tower-based systems like this can provide short- and long-term datasets to assess genotypic and/or management responses to the environment, and ultimately enable the spectral prediction of resource-use efficiency, stress resilience, productivity and yield.

## Background

Plant phenotyping requires tools that can quantify plant structure, function, and their response to environmental conditions with high precision, high throughput, and across scales from organs to whole plants and canopies [1, 2]. There is a myriad of applications for plant phenotyping among plant physiologists, plant breeders, ecologists, and land managers. Importantly, trait data acquired through plant phenotyping can inform species and genotype selection to increase resilience to stresses such as drought and overall adaptation to future climates, thereby assisting crop breeding for future global food demands [3, 4]. Thus, there is great interest in advancing high-throughput plant phenotyping tools to monitor the variation of vegetation across genotypes in response to the environment.

Despite much progress, plant phenotyping remains a bottleneck and lags behind our ability to characterize plant genomes, hampering progress in research and breeding [5, 6]. This is because traditional phenotyping methods are often low-throughput (i.e., labor intensive and time consuming), limited in application to large areas, potentially subjective, and/or destructive—leading to severe limitations in sampling scale and frequency [7]. In recent years, plant phenotyping has advanced tremendously, leading to high-throughput phenotyping methods such as RGB (red, green, blue) imaging, thermal imaging, and hyperspectral remote sensing that are scalable and non-destructive [8]. Instruments designed for this purpose have been deployed on various platforms [9], including handheld devices, ground-based vehicles [10], tower-based systems [11–13], unoccupied aerial vehicles (UAVs) [14, 15], piloted aircraft [16, 17], and satellites [18, 19]. While each platform provides promising applications for high-throughput phenotyping, each has spatial, temporal (intermittent vs automated deployment), spectral (multi- vs hyperspectral) limitations, and require a wide range of necessary corrections for adequate interpretation (e.g., geometric, radiometric, atmospheric, etc.).

This paper presents TSWIFT (Tower Spectrometer on Wheels for Investigating Frequent Timeseries), a mobile tower-based hyperspectral remote sensing system suited for short- and long-term field deployment. A key advantage of a tower-based system is the ability to collect data continuously and automatically in nearly any environment, thus reducing personnel requirements while increasing spatial resolution and robustness to variable weather conditions (clear and cloudy sky conditions). The system described in this study acquires point measurements of specific targets in about five seconds, enabling data acquisition at high frequency across diurnal and seasonal temporal scales. It collects co-located RGB images and hyperspectral reflectance data in the visible and near-infrared (NIR) regions, and can also resolve the far-red solar-induced fluorescence (SIF) signal. Combined, these capabilities allow for the assessment of physical attributes and physiological variation from simple vegetation indices or from machine learning techniques [20–22].

High spectral resolution enables qualitative assessment of vegetation function using simple vegetation indices or by exploiting nuances across the entire spectrum. For this paper, we focus on three vegetation indices sensitive to structural or physiological dynamics, but acknowledge many other vegetation indices that could be explored for assessing various traits and features [23–25]. For structural dynamics, we focus on the normalized difference vegetation index (NDVI, commonly used to infer canopy greenness), leaf area index (LAI), and light absorption [26, 27]. These structural measures are generally associated with longer term variations throughout the growing season, limiting applications in monitoring physiologically dynamic processes. For physiological dynamics, we focus on the photochemical reflectance index (PRI) and SIF. PRI is sensitive to variations in xanthophyll content, a key element of excess energy dissipation often used as a proxy of photosynthetic activity [28]. Temporal scale must be considered when interpreting PRI, because it is sensitive to both short-term xanthophyll cycle variation and long-term carotenoid/chlorophyll pigment pools [29, 30]. SIF is derived from the reemission of absorbed photons via chlorophyll under sunlight, and provides an estimate of photochemical activity and energy dissipation [22, 31, 32]. Similar to PRI, SIF requires temporal context in linking signal variation to physiological mechanisms [33, 34]. Beyond simple vegetation indices, hyperspectral data enables the use of full-range spectra for partial least squares regression (PLSR) models to estimate a suite of parameters associated with photosynthetic metabolism [35] and leaf biochemical status [36]. The combination of simple vegetation indices and full range hyperspectral data provides a powerful tool for phenotyping plant structure and function with high throughput—over time and across many genotypes.

The objective of this paper is to present the technical details and ideas for similar design concepts of a mobile tower-based remote sensing system, TSWIFT, that generates continuous and automated point hyperspectral reflectance and SIF data of designated targets. The primary purpose of this system is to capture short-term (diurnal) and long-term (seasonal) variation of vegetation function, but here we focus on its application for high-throughput phenotyping. In this study, we demonstrate TSWIFT's ability to phenotype variation of phenology and drought resilience across diverse genotypes of common bean (*Phaseolus vulgaris* L.) with contrasting heat and drought adaptation. To accomplish this, we show remotely sensed proxies of vegetation structure (e.g., NDVI) and function (e.g., PRI and SIF) as a proof-of-application for a tower-based hyperspectral remote sensing system.

## Materials and methods

### TSWIFT system design

**Fig. 1:**
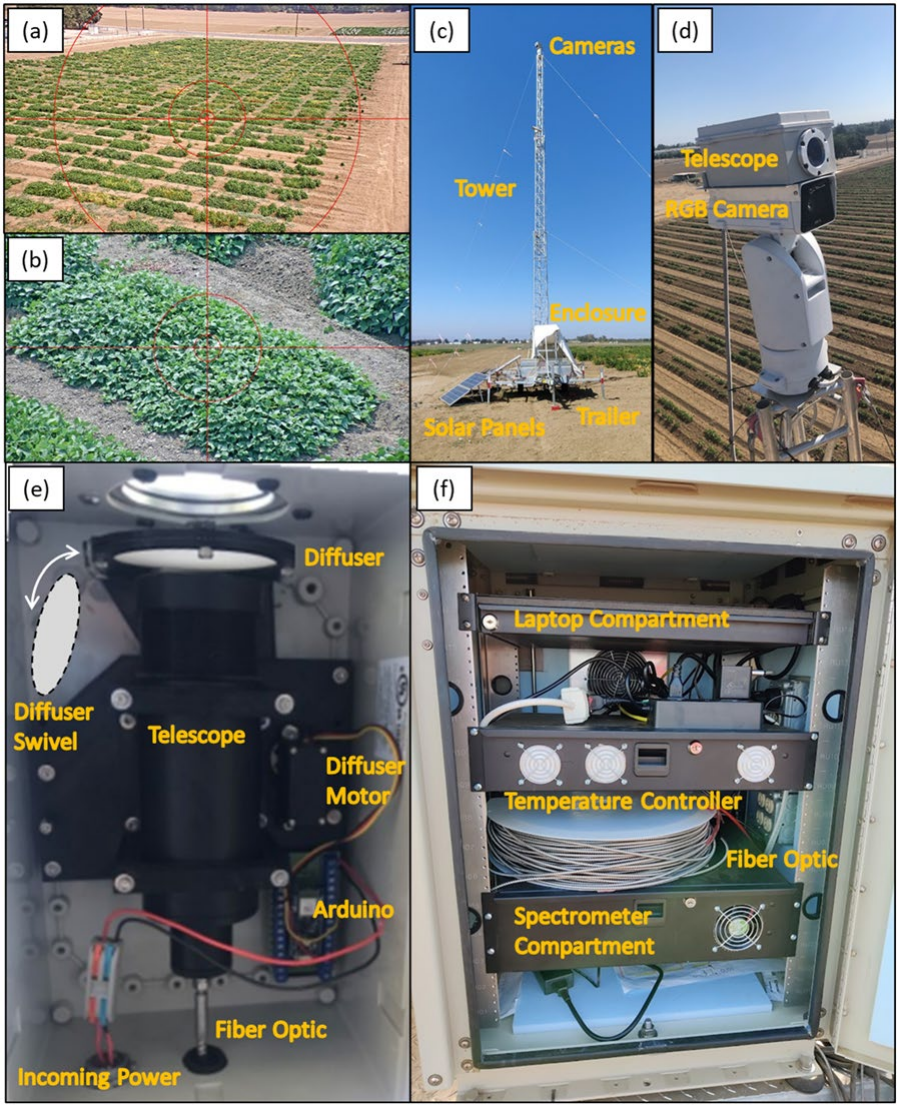
Images of a section of the field site (**a**), an example RGB target image (**b**), the full tower and TSWIFT system (**c**), the RGB camera and 2D scanning telescope enclosure (**d**), interior of the 2D scanning telescope enclosure (**e**), and interior of the temperature-controlled enclosure (**f**)

TSWIFT is a tower-based spectrometer and RGB camera system using scanning point measurements of user-specified targets (i.e., vegetation, ground, sky, etc.) for monitoring hyperspectral reflectance in the visible and NIR regions and capability to resolve far-red SIF, coupled with RGB imagery (Fig. 1). This instrument extends the original design of PhotoSpec [37], but with modifications suited more for high-throughput phenotyping applications. The weather protected RGB camera (AXIS Q8685-E PTZ Network Camera, Axis Communications AB, Lund, Sweden) enables 360° pan, a ground-to-sky view from −45° to 90°, and 30 × optical zoom for sample targeting and spot RGB imagery (Fig. 1d). Mounted on top of the RGB camera is an enclosed co-located 2D scanning 2-inch aperture telescope unit (Thorlabs Inc., NJ, USA) designed to collect radiance/irradiance from any specified target (Fig. 1d, e). The telescope has a field of view (FOV) of 0.7° to enable spot targeting of individual plants. Colocation of the RGB camera and telescope was completed by projecting a laser pointer out of the telescope and aligning it with the center point of the RGB camera at a distance of ~ 20 m. The telescope enclosure also consists of an opal diffuser (~ 12% transmission efficiency) on an Arduino powered motor that enables the diffuser to swivel in front of the telescope during hemispherical irradiance (i.e. incoming radiation) measurements, and away from the telescope during target radiance measurements to maintain FOV of 0.7°.

Connected to the telescope is a fiber optic cable with a stainless-steel jacket that extends to the base of the tower and into the temperature-controlled enclosure (Fig. 1f). Here the fiber connects to a quad-furcated fiber bundle enabling the connection of up to four spectrometers. For this setup, we connected two thermally stabilized spectrometers (Ocean Insights, FL, USA): the QE Pro for measuring SIF (729 to 784 nm, full width at half maximum [FWHM] = 0.3 nm); and the FLAME for hyperspectral reflectance (338 to 1022 nm, FWHM = 1.2 nm). Both spectrometers are housed in a small thermally controlled enclosure (described below) maintained at 25 °C (Fig. 1f). The spectrometers and the RGB camera are connected and controlled by a field laptop (Latitude 5400, Dell, TX, USA) located inside the temperature-controlled enclosure. The field laptop is connected to a mobile internet hotspot (MiFi 8800L, Verizon, NY, USA) for data acquisition, remote access to view and control TSWIFT, and optional data upload.

The temperature-controlled enclosure prevents overheating of the laptop and ensures stable temperature control of the spectrometers. The enclosure is a NEMA 3R Radio Cabinet Enclosure (Aluma Tower Company INC., FL, USA) located on a portable tower trailer (TM 12, Aluma Tower Company INC., FL, USA). This enables the tower to be quickly moved to different locations for short- or long-term monitoring. The portable tower can extend to 15.24 m (50 ft) and is secured guy wires, as was done for the experiment described here (Fig. 1c). The enclosure was powered by a 1000 W 24 V solar array for remote locations. Alternative configurations could include a permanent tower, or a fixed AC or generator to provide electrical power.

### TSWIFT Data collection

TSWIFT allows for user specified targets ranging from a pan of 0 to 360° and tilt from -45 to 90°. Target duration for measurements can also be specified, where the longer the duration, the more repeat measurements will occur. Therefore, the number of targets versus measurement duration will depend on the research objectives, and will influence the quantity and frequency of repeat measurements (e.g., more targets or longer duration leads to less frequent repeat measurement cycles; less targets or shorter duration leads to more frequent repeat measurement cycles).

At each user specified target location, multiple spectral measurements will occur over the user specified duration, after which the target changes. During each measurement, spectral retrieval was optimized for both spectrometers using automatic integration time optimization. Each measurement (both target and sky) integration time is automatically adjusted to achieve signal strength of approximately 80% to saturation to maximize signal to noise ratio.

### TSWIFT data processing

**Fig. 2:**
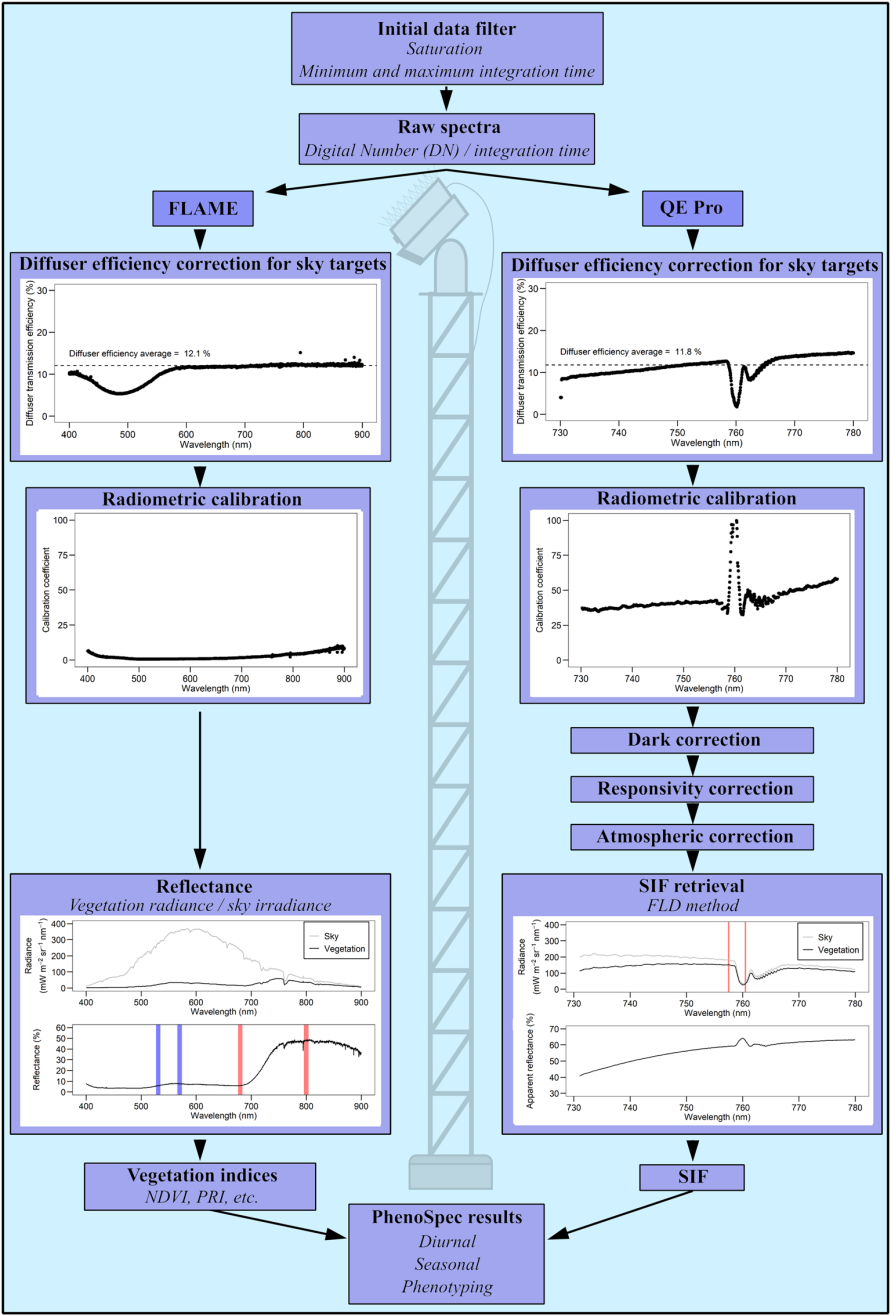
TSWIFT data processing workflow. See “TSWIFT data processing” section for details. For the Reflectance box, radiance represents the incoming sky irradiance and reflected target vegetation radiance. Reflectance is the target vegetation radiance divided by sky irradiance. Vertical lines represent the reflectance bands used for vegetation index calculations for NDVI (red; Eq. 1) and PRI (blue; Eq. 2). For the SIF retrieval box, radiance represents the incoming sky irradiance and reflected target vegetation radiance. Apparent reflectance is the target vegetation radiance divided by sky irradiance. Vertical red lines represent the wavebands used to calculate SIF using the Fraunhofer Line Depth (FLD) method (Eq. 3)

The full data processing workflow is shown in Fig. 2. All target scans were saved as daily NetCDF (.nc) files containing target names, datetime stamps, integration time, camera position (pan and tilt), and the full the hyperspectral data from each spectrometer. In the following sections, we discuss the processing procedure from raw data through to reflectance data and SIF retrieval.

**Fig. 3:**
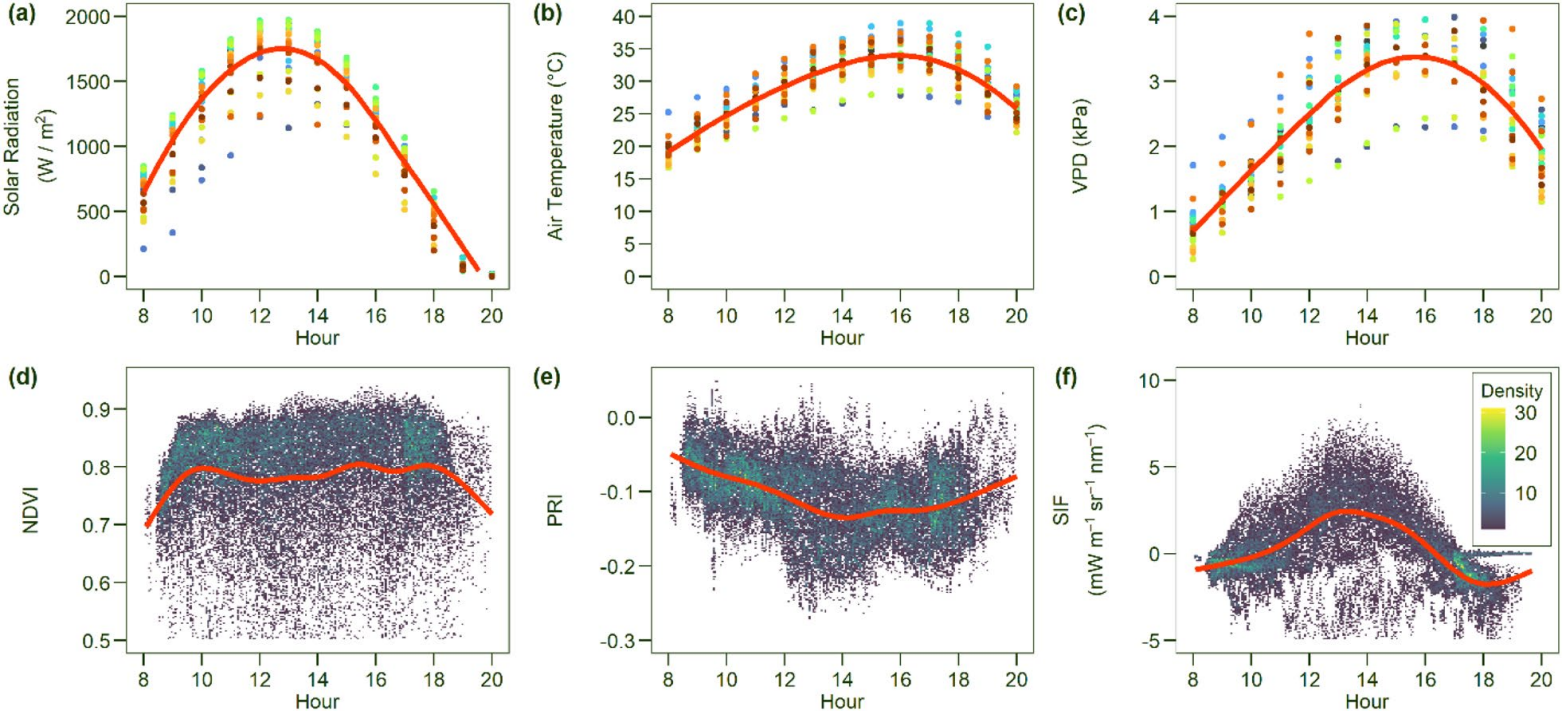
Diurnal variation of hourly solar radiation (**a**), air temperature (**b**), and vapor pressure deficit (VPD; **c**) where colored points represent different days. Diurnal variation of vegetation NDVI (**d**), PRI (**e**), and SIF (**f**) of all acquired targets where colored points represent point density (bin size is 200). Red line represents LOESS fitting to highlight overall diurnal pattern. Dates used are from July 25 to August 5, which serves as a baseline prior to drought treatment response

#### Initial data filtering and raw spectra

Initial data filtering is done to exclude poor quality data based on saturation and integration time. Saturation was screened by removing any measurement where the hyperspectral data reaches a digital number (DN) of 65,535 and 200,000 for the FLAME and QE Pro spectrometers, respectively, which are the maximum saturation limits of the spectrometers. Depending on light intensity, a range of integration times (0.05 to 60 s) is chosen to maximize signal:noise ratio. Data with integration times outside of this range was excluded due to a low signal resulting from either an overly short integration time or a long integration time, which typically was a result of low irradiance/reflected radiance. To account for varying integration time per measurement from the automatic optimization, all raw DN were divided by their respective integration times.

#### Diffuser transmission efficiency

All “sky” measurements used a diffuser to capture hemispherical irradiance (i.e. incoming radiation), which is used later to calculate vegetation reflectance. For computing reflectance, diffuser transmissivity must be known. We estimated diffuser transmission efficiency in the field at 13 h by pointing the camera straight up at 90°, acquiring repeat measurements with scan times of ~ 1 s for 30 min, and periodically (every ~ 5 min) removing or replacing the diffuser in the telescope FOV. For the FLAME, the diffuser transmission efficiency was estimated for each wavelength as the ratio of measured irradiance with and without the diffuser. For the QE Pro, an average diffuser efficiency from the spectra was used for all wavebands (11.8%). We applied a wavelength and spectrometer specific diffuser efficiency to correct all “sky” irradiation measurements which used the diffuser.

#### Radiometric calibration

The QE Pro and FLAME spectrometers require radiometric calibrations to convert units from digital numbers (DN) to radiance (mW cm^−2^ sr^−1^ nm^−1^). We performed radiometric calibrations in the field by taking “sky” measurements with the camera pointing straight up at the sky (90°) with concurrent measurements from a radiometrically calibrated field spectrometer (350 to 2500 nm) (HR-1024i, Spectra Vista Corporation, Poughkeepsie, New York, USA) [36, 37]. The field spectrometer with a 4° FOV, was pointed at a calibrated Spectralon diffuse reflectance standard (Labsphere Inc., NH, USA), which is highly Lambertian, and 99% reflective over a wavelength range from 250 to 2500 nm. The QE Pro, FLAME and HR-1024i spectrometers took repeat measurements of their respective sky or reflectance standard every minute for 1 h from 12 to 13 h under clear sunny conditions. Ideally, field calibration measurements should take place as frequently as possible (beginning and end of deployment at a minimum) to check for drift in the spectrometers.

Calibration coefficients were determined for each spectrometer (QE Pro and FLAME) by relating raw DN with the HR-1024i radiance through fitting a line through the calibration data with an intercept of zero. For the FLAME (FWHM = 1.2 nm), the calibration coefficient for each respective wavelength was then applied to all radiance and irradiance measurements taken over the course of the experiment to radiometrically calibrate all raw DNs to radiance units. For the QE Pro (FWHM = 0.3 nm), which was much finer spectral resolution compared to the HR-1024i, we used a mean calibration coefficient across all wavelengths.

#### Hyperspectral reflectance (FLAME spectrometer)

Spectral reflectance for each target was determined by dividing target radiance by sky irradiance (measured closest in time to the target radiance; within 35 s), corrected for the diffuser transmission efficiency (Fig. 2). We used average reflectance of a 10 nm window centered at 680 (R_Red_) and 800 nm (R_NIR_), and 531 (R_531_) and 570 nm (R_570_) for NDVI and PRI, respectively.

1 $$\mathrm{NDVI}= \frac{(\mathrm{R}_\mathrm{NIR}- \mathrm{R}_\mathrm{Red})}{(\mathrm{R}_\mathrm{NIR}+ \mathrm{R}_\mathrm{Red})}$$

2 $$\mathrm{PRI}= \frac{(\mathrm{R}_\mathrm{531}- \mathrm{R}_\mathrm{570})}{(\mathrm{R}_\mathrm{531}+ \mathrm{R}_\mathrm{570})}$$

#### Solar induced fluorescence retrieval (QE Pro spectrometer)

Following radiometric calibration of the QE Pro, an electronic dark correction, responsivity correction, and atmospheric correction was applied, see Marrs et al. [38] for details. For the electronic dark correction, the fiber optic was disconnected from the QE Pro and replaced with a black cap with the QE Pro located in a dark enclosure. Here the electronic dark responsivity was obtained, which was applied for all measurement scans on a per waveband basis, with the assumption of a stable dark correction as the QE Pro, spectrometer enclosure, and outdoor enclosure were all temperature controlled. For the atmospheric correction, we used meteorological data from the nearest Automated Surface Observing Systems (ASOS; https://www.weather.gov/asos/asostech), located about 500 m West of TSWIFT in Davis, CA (station code: EDU). This atmospheric correction accounts for temperature, air pressure, humidity, and path length to each target.

Given the technical specifications of this setup, many different SIF retrievals could be performed. However, here the SIF retrieval was performed using the Fraunhofer Line Depth (FLD) method [39] using an in-filling in the atmospheric O_2_-A absorption feature. This utilized a combination of spectral radiance (L) and irradiance (E) at 757.5 and 760.5 nm, following the protocol outlined in Marrs et al. [38]:

3 $$\mathrm{SIF}= \frac{\left(\mathrm{E}_\mathrm{757.5}\times \mathrm{L}_\mathrm{760.5}\right)- \left(\mathrm{L}_\mathrm{757.5}\times \mathrm{E}_\mathrm{760.5}\right)}{(\mathrm{E}_\mathrm{757.5}- \mathrm{E}_\mathrm{760.5})}$$

### Study site and design

The experimental design consisted of a diverse multi-parent breeding population of 300 common bean genotypes (*P. vulgaris*) [40]. All genotypes were grown in the field at the Plant Sciences Field Facility of the University of California, Davis (38.534^o^N, 121.775^o^W) from June to October 2021 in designated irrigated and terminal drought treatments with three replicate plots each per genotype. Each plot was 3.05 m long (N-S) and 1.52 m wide (E-W) with two planted rows spaced 66 cm apart, and separated from adjacent plots by lanes 1.22 m long (in the N-S direction) or 1.52 m wide (in the E-W direction) (see Fig. 1a for example of plot arrangement). Both treatments were watered using aboveground drip irrigation during initial growth, then switched to belowground 50 cm depth irrigation after stand establishment. We applied terminal drought by stopping irrigation to the drought treatments on July 26. To initiate senescence, irrigation for the control plots was terminated on September 1.

TSWIFT was set up approximately in the middle of the field. With a tower height of 15.24 m, we limited the viewing radius of the system to 72.6 m (250 ft). This enabled the measurements of 720 plot targets (360 per treatment) with 175 genotypes represented in both treatments. The system had a 5 s measuring time per target. A sky irradiance measurement occurred every 15 targets (within 35 s). Each complete scan cycle took approximately 3 h resulting in about three revisit intervals for a single day.

### Meteorological data

Hourly meteorological data was downloaded from the California Irrigation Management Information System (CIMIS, California Department of Water Resources). CIMIS station ID 6 was located in Davis, CA about 250 m North-West from TSWIFT. Daily mid-day means of solar radiation, air temperature, and vapor pressure deficit (VPD) were determined using an afternoon period from 11 to 16 h. Daily total precipitation was also obtained from the CIMIS station. Daily mean particulate matter (PM2.5) data was downloaded from the US Environmental Protection Agency (EPA) Air Quality System Data Mart. The nearest monitoring station was located in Davis, CA about 150 m North-East from TSWIFT.

### Data analysis

Data was processed in R [41]. To evaluate diurnal variation, we focused on data from July 25 (approximately stage R5: Pre-flowering) to August 5 (stages R7-R8: pod formation-beginning pod fill) [42]. This period was selected to correspond with considerable canopy cover, flowering, and early pod fill, and maximize sample size prior to the effects of drought treatment. To evaluate seasonal variation, we used daily mid-day means from 11 to 16 h. This solar noon window was chosen to maximize sensor signal-to-noise ratios and limit the viewing geometry phase angles (see “Evaluating sun/sensor angular effects” section). To evaluate genotypic differences for the vegetation indices, we calculated the relative percent difference between drought and control treatments for NDVI, PRI, and SIF for each genotype constrained with daily mid-day means from 11 to 16 h (relative % difference = 100 [Drought − Control]/|Control|). To explore the extent of variability across the full range of the FLAME hyperspectral dataset, we calculated coefficients of variation (CV; ratio of standard deviation to mean) for all daily mid-day data across treatments and genotype. For genotypic CV, we limited dates to August 1 to August 5 and control treatment only, to highlight genotypic variation prior to flowering. For treatment CV, we limited dates to August 25 to August 30, about 1 month after drought treatment. For growing season CV from June 25 to October 1, we only evaluated control treatments to minimize genotypic variation of drought resilience.

### Evaluating sun/sensor angular effects

To evaluate the effects of solar and viewing sensor geometries on the tower-based hyperspectral reflectance data, vegetation indices, and SIF, we utilized soil target data prior to vegetation growth (June 1 to 15). Here, we assumed soil to be a flat homogenous surface and that any variation observed is due to solar and viewing sensor geometry. We calculated the phase angle, which is the angle at a given point between the sun and sensor [43, 44]. Phase angle considers the relative azimuth angle (RAA) between the viewing azimuth angle (VAA) from the sensor and solar azimuth angle (SAA) relative to north in a clockwise direction. In addition, phase angle also incorporates solar zenith angle (SZA) and viewing zenith angle (VZA).

4 $$\mathrm{RAA}=\mathrm{VAA}-\mathrm{SAA}$$

5 $$\mathrm{Phase~angle}= {\mathrm{cos}}^{-1}\left[\begin{array}{c}\mathrm{cos}\left(\mathrm{SZA}\right)\times \mathrm{cos}\left(\mathrm{VZA}\right)+\\ \mathrm{sin}\left(\mathrm{SZA}\right)\times \mathrm{sin}\left(\mathrm{VZA}\right)\times \mathrm{cos}\left(\mathrm{RAA}\right)\end{array}\right]$$

## Results

### Performance of TSWIFT at the diurnal scale

TSWIFT provides continuous monitoring at the diurnal scale (Fig. 3). Here, we use NDVI, PRI, and SIF to evaluate the diurnal patterns observed. NDVI shows a relatively stable pattern throughout the day with the largest variation in the early morning and late evening (Fig. 3d). In contrast, PRI shows a “U” shaped pattern with lowest values towards midday (Fig. 3e). SIF on the other hand, shows a bell-shaped curve with highest values towards midday (Fig. 3f).

### Performance of TSWIFT at the seasonal scale

**Fig. 4:**
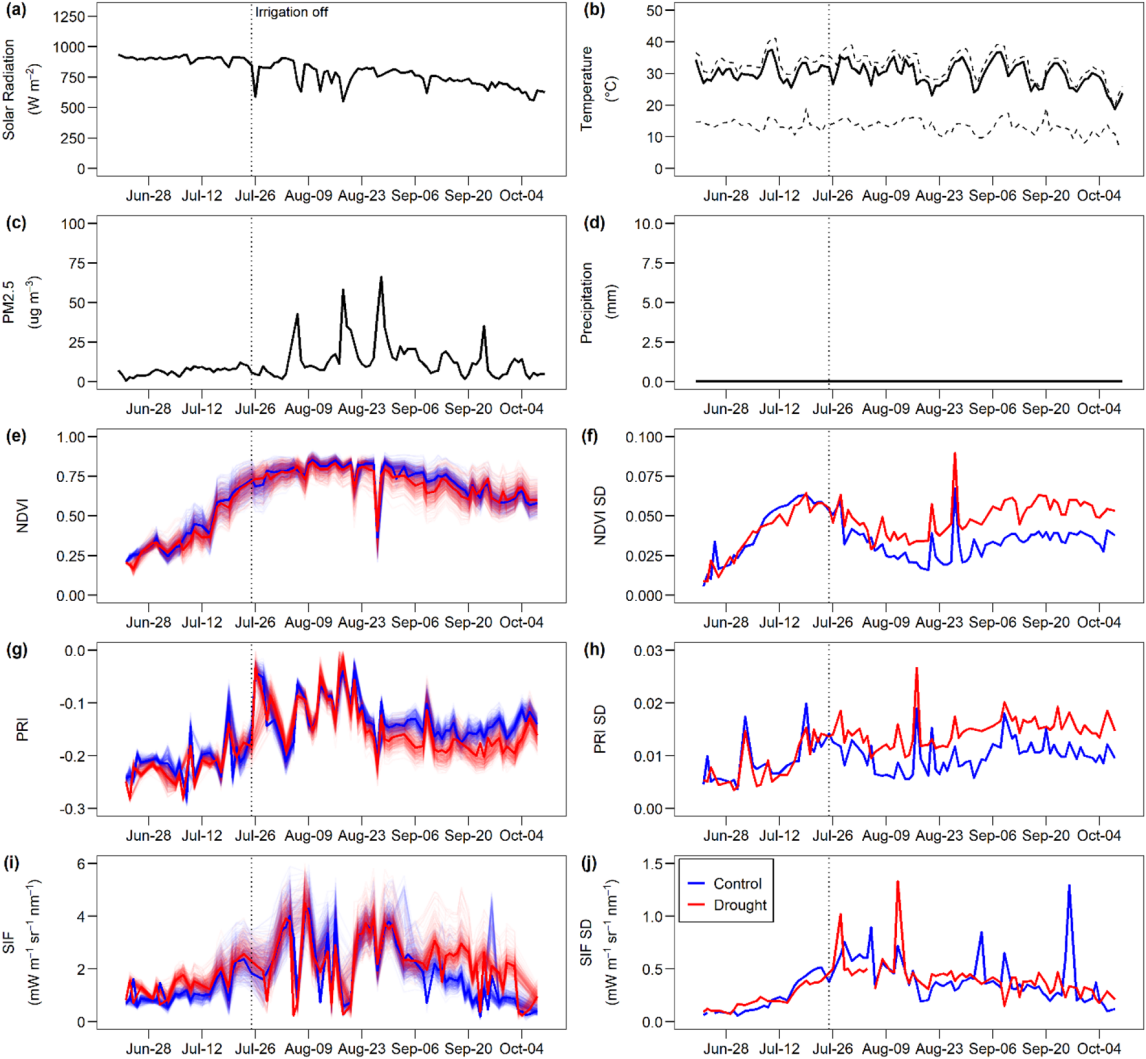
Daily mid-day mean (11 h to 16 h) of solar radiation (**a**), daily mid-day mean, minimum and maximum air temperature (**b**), particulate matter 2.5 concentrations (c), precipitation (**d**), and the daily mid-day (11 h to 16 h) means and standard deviations (SD) of NDVI (**e**, **f**), PRI (**g**, **h**), and SIF (**i**, **j**) per treatment. Thin lines represent each plot target and thick line represents overall treatment means

Continuous monitoring at the seasonal scale enables long-term assessment of vegetation structure and function over the growing season (Fig. 4). For NDVI, there is an increase at the start of the growing season, which stabilizes and remains mostly constant through August, eventually showing a minor decline later in the season (Fig. 4e). PRI also showed seasonal variation with an increase early in the growing season and a decrease later in the season in late August (Fig. 4g). However, PRI is more variable, especially throughout August. Similar to PRI, SIF shows a seasonal pattern with an increase early in the growing season, but a more gradual decline later in the season in September (Fig. 4i). SIF was also highly dynamic in August, consistent with more variable sky conditions during this time. To highlight variation across genotypes, we show a timeseries of the standard deviation (SD) of NDVI, PRI and SIF across treatments (Fig. 4f, h, j). At the beginning of the season, before drought treatment was applied, the SD between treatments were similar in all vegetation indices. After the onset of terminal drought, the SD diverge between control and drought treatments – exhibiting generally higher SD in the drought treatments in NDVI and PRI as the season progresses. SIF SD were similar between treatments throughout the growing season.

### High-throughput phenotyping of drought resilience across genotypes

**Fig. 5:**
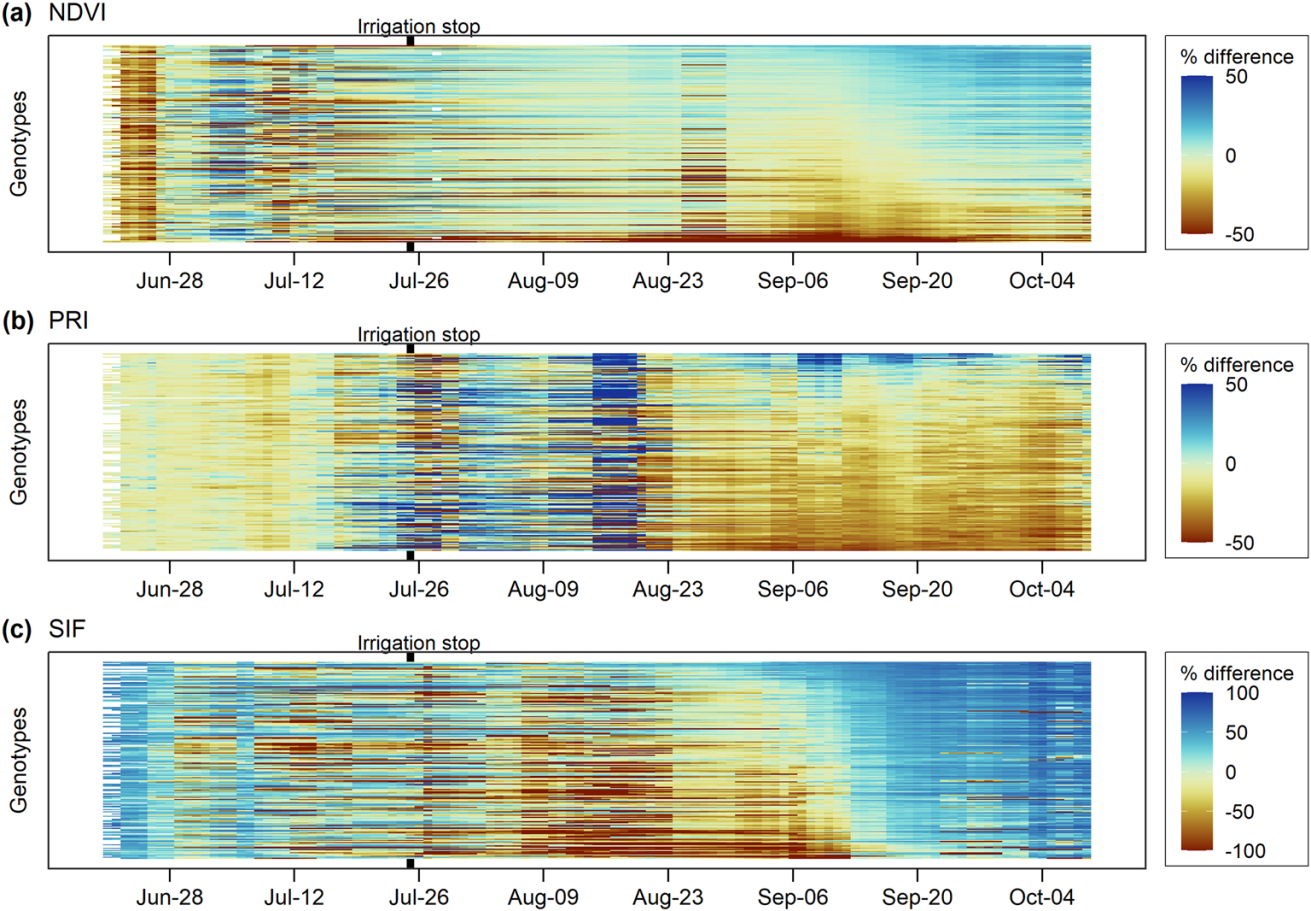
Visualization of genotypic variation (n = 75) of the relative percent difference between drought to control treatment per genotype for daily noontime mean data (11 h to 16 h) for NDVI (**a**), PRI (**b**) and SIF (**c**). Note different color scale range for each panel. Relative % difference = 100 [Drought−Control]/|Control| where red represents lower values for drought relative to control and blue represents higher values for drought relative to control treatments

Continuous and automated high-throughput phenotyping can highlight genotypic variation of drought resilience across genotypes. Here, we determined the relative percent difference between control and drought treatments for NDVI, PRI, and SIF to show divergent treatment responses over time across genotypes (Fig. 5). Relative percent difference for NDVI, PRI and SIF all show a large range of variation across genotypes. NDVI showed large genotypic variation in early September (Fig. 5a). PRI exhibited variation throughout the entire growing season with the largest relative percent differences, generally negative, occurring in August and September (Fig. 5b). In contrast, SIF had the largest variation of relative percent difference throughout August, which was negative, but became positive in September (Fig. 5c). Although the relative percent difference in both PRI and SIF between genotypes spiked immediately after the onset of terminal drought, this was likely driven, at least in part, by a brief decline in incident irradiation at that time (Fig. 4a).

## Discussion

Proximal remote sensing systems like the TSWIFT are valuable for ecophysiological applications by providing continuous and automated monitoring of vegetation spectra. From spectra, vegetation structure and function can be inferred to assess the short- and long-term dynamics of vegetation across environmental conditions. In addition to vegetation monitoring, a point scanning system enables applied use for high-throughput phenotyping across genotypes for select traits such as drought resilience. Here we highlight the potential of the mobile tower-based TSWIFT system, which can evaluate genotypic/management variations of vegetation response to short- and long-term environmental dynamics, with applications for high-throughput phenotyping.

### Diurnal applications

At the diurnal scale, dynamic physiological mechanisms regulate light energy balance, carbon fixation, and water loss driven by environmental variation such as incoming radiation, temperature, and VPD. TSWIFT demonstrated that physiologically sensitive vegetation indices like PRI (Fig. 3b) and SIF (Fig. 3c) can track diurnal variation. Diurnal shifts in PRI reflect pigment conversion in the xanthophyll cycle between violaxanthin, antheraxanthin and zeaxanthin [28]. Xanthophyll cycle conversion is linked to non-photochemical quenching (NPQ) and heat excess energy dissipation to regulate light energy balance when photochemistry saturates or becomes limited [45]. PRI has been utilized as a proxy of photosynthetic activity and light-use efficiency (LUE) [46, 47]. Thus, the observed decrease in diurnal PRI represents decreasing LUE near solar noon (Fig. 3b). SIF represents another dynamic aspect in regulating light energy balance via the emission of chlorophyll fluorescence [31]. Diurnally, SIF increases near mid-day (Fig. 3c), which is primarily a response of SIF to absorbed photosynthetically active radiation (APAR), and has been shown in many previous studies [33, 48]. In contrast to the physiological vegetation indices, the structure-based NDVI, showed minimal diurnal variation throughout most of the day (Fig. 3a). Being structurally sensitive and often considered a proxy of APAR and LAI [26], NDVI is less dynamic especially under clear sunny conditions. While we only show three vegetation indices here for demonstration purposes, we highlight the potential of the TSWIFT to assess highly dynamic processes, to aid in real-time management and decision making, or to inform breeding applications targeting the genotypic optimization of physiology at certain times of the day.

### Seasonal applications

At the seasonal scale, plants undergo structural and physiological changes to maximize growth and productivity. Here, over the course of the growing season, NDVI initially increased as the plants emerged from seed and underwent a green up period (Fig. 4e), which is associated with increasing biomass and LAI [49, 50]. Towards the end of the growing season, NDVI slowly declined as senescence occurred. In contrast, PRI and SIF showed a more gradual increase and decrease throughout the season, suggesting periods of peak photosynthetic activity in August, but also a strong response to incoming light (Fig. 4g, i). At this temporal scale, PRI is likely representing changes in a combination of the xanthophyll cycle composition and carotenoid/chlorophyll pigment pool ratio [51]. Both of these physiological mechanisms are associated with LUE and NPQ [45], suggesting that the increasing PRI is indicative of increasing photosynthetic activity and LUE as leaves develop their photosynthetic capacity [52]. SIF on the other hand represents the re-emission of photons from photosystem II, and is expected to increase with light, photochemical activity and total chlorophyl content [31]. During leaf development, an increase in chlorophyll content enhances light absorption and therefore total emission of chlorophyll fluorescence [53, 54]. At these longer time scales over the growing season, SIF is generally closely associated with gross primary productivity [22, 33]. SIF was higher in the drought treatment in September (Fig. 4i), presumably representing increased chlorophyll fluorescence due to constraints on photochemical quenching associated with closing stomata, or a shift to a ‘photoinhibitory’ phase when NPQ is saturated [55]. However, understanding the mechanisms leading to higher SIF in drought vs. control towards the end of the season were beyond the scope of this study, and should not be interpreted as such.

We note that the NDVI, PRI, and SIF signal may be confounded during smokey air conditions, with sharp increases/declines during periods of lower incoming radiation and high PM2.5, which occurred multiple times in August (Fig. 4c, i). Comparing the vegetation indices, PRI and SIF were more dynamic and decreased prior to changes in NDVI (Fig. 4), suggesting that the physiological changes occur prior to any detectable changes in canopy greenness. Therefore, a combination of vegetation indices like NDVI, PRI, and SIF may provide complementary information on canopy structure and photosynthetic activity over the growing season representing different aspects of plant growth and development [56, 57].

### High-throughput phenotyping

The high spatial (30 to 60 cm) and temporal resolution (sub-hourly to daily to seasonal) provided by the tower system presented here enables generation of a continuous dataset for high-throughput phenotyping. As an example, we visualize the standard deviation (SD, Fig. 4) and relative percent difference between treatments across genotypes in NDVI, PRI, and SIF (Fig. 5). For SD of NDVI and PRI, drought was generally higher than control treatments, indicative of greater genotypic variation in drought response (Fig. 4a, b). In contrast, SIF SD was largely similar between treatments (Fig. 4j), suggesting similar treatment variation of SIF signal across all genotypes. For relative percent difference, NDVI generally had the largest differences by late August (Fig. 5a) with large genotypic variation due to differences in the senescence stage of the different genotypes included in the field trial [58]. PRI showed genotypic variation across most of the season with the most pronounced differences occurring in late August (Fig. 5b). This may highlight differences in both the xanthophyll cycle representing short-term variation in response to drought and long-term variation in pigment pools associated with growth stage [59–62]. Interestingly, SIF showed the greatest differences and genotypic variation throughout August during the initial drought period (Fig. 5c). Here, the relative values between treatments showed drought having lower SIF than the control. Then when the control treatments begin to senesce in September, control SIF was lower than the drought SIF (Fig. 5c). This was unexpected as stressed plants tend to exhibit a lower SIF signal relative to unstressed plants [33, 63, 64]. We suspect that multiple factors in canopy structure (e.g., wilting) and leaf physiology (e.g., stress response and senescence) may influence the drought response of SIF [65]. The high variation of SIF, NDVI, and PRI across genotypes and time are likely associated with genotype-specific drought response and resilience [63, 66]. With a hyperspectral system, a number of plant traits can be inferred, allowing for diagnosis of different physiological mechanisms among genotypes and their variability in environmental responses.

### Hyperspectral applications

**Fig. 6:**
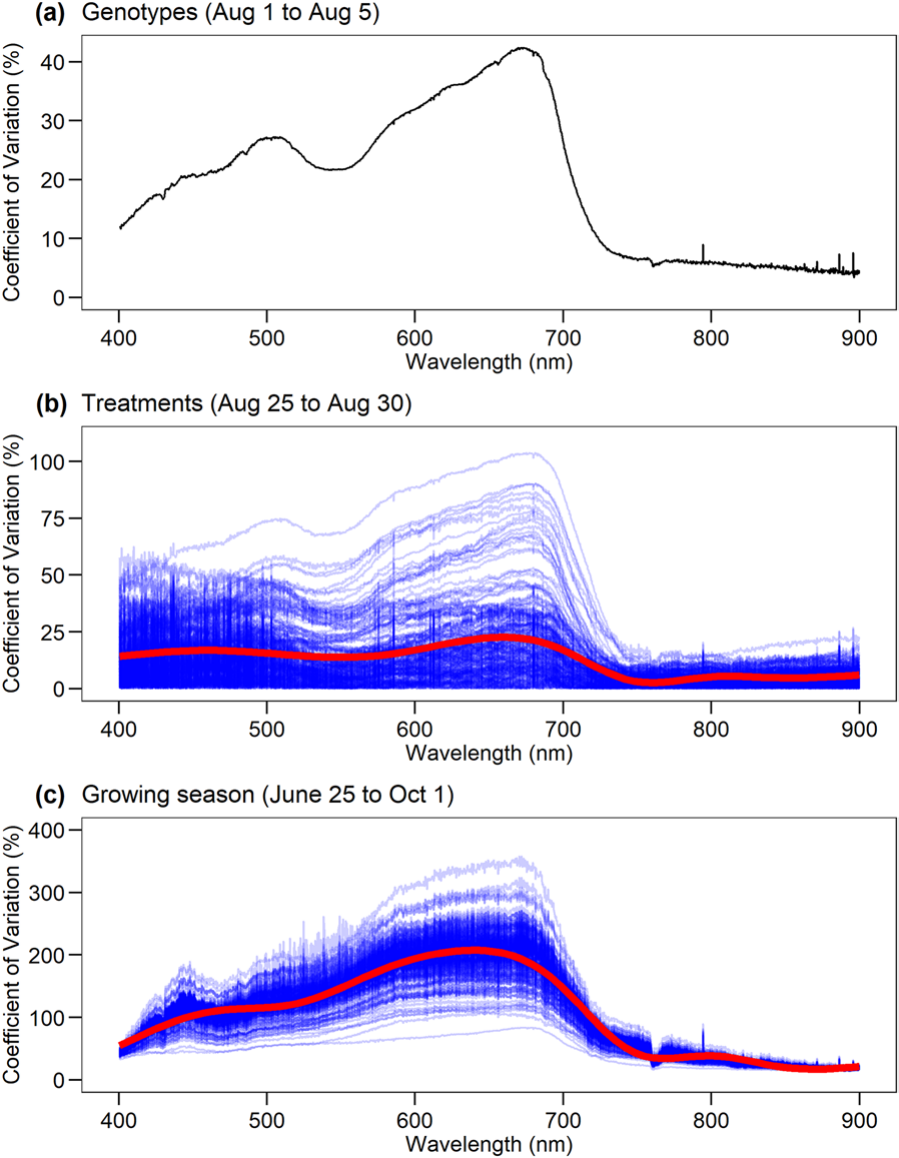
The coefficient of variation (CV) across the FLAME hyperspectral data from noontime means across control treatment genotypes (**a**), between treatments (**b**), and over the growing season of control plots (**c**). Blue lines represent each individual genotype, and red line represents overall mean. Note different y-axis range

The examples shown in this paper focused on structural (NDVI) and physiological (PRI and SIF) vegetation indices. However, with the FLAME spectrometer, TSWIFT acquires visible and NIR hyperspectral reflectance at a ~ 2 nm spectral resolution (400 to 900 nm). This enables more powerful statistical techniques such as partial least squares regression, principal components analysis, independent component analysis, singular-value decomposition, and other machine learning techniques for model development using the full hyperspectral range to predict an array of plant traits [21, 67–70]. These approaches have shown potential for estimating structural (e.g., leaf mass per area), biochemical (e.g., nitrogen, carbon, and phosphorus content), and physiological traits (e.g., photosynthetic parameters, and pigment composition) [35, 69, 71–75]. They may also have mechanistic biophysical ties to specific spectral regions that are reflect leaf surface properties and internal structure, which ultimately influence physiological function [76]. To explore the relatively variable spectral regions of our hyperspectral data due to treatment and genotypic contrasts, we determined the coefficient of variation (CV) and found that the visible region (500 to 700 nm) and red edge (680 to 730 nm) are sources of high variability across genotypes, between well-watered and terminal drought treatments, and across the entire growing season (Fig. 6). This is notable as the visible region is sensitive to chlorophyll and carotenoid pigments and the red edge is often used to assess chlorophyll content, while the NIR has weaker absorption features mainly influenced by leaf structure and water absorption features [20, 77]. While these statistical approaches show great promise for estimating plant traits, much work is needed to evaluate their application and robustness over different spatial and temporal scales and across years with respective validation data, which is beyond the scope of this paper.

### Limitations and considerations

**Fig. 7:**
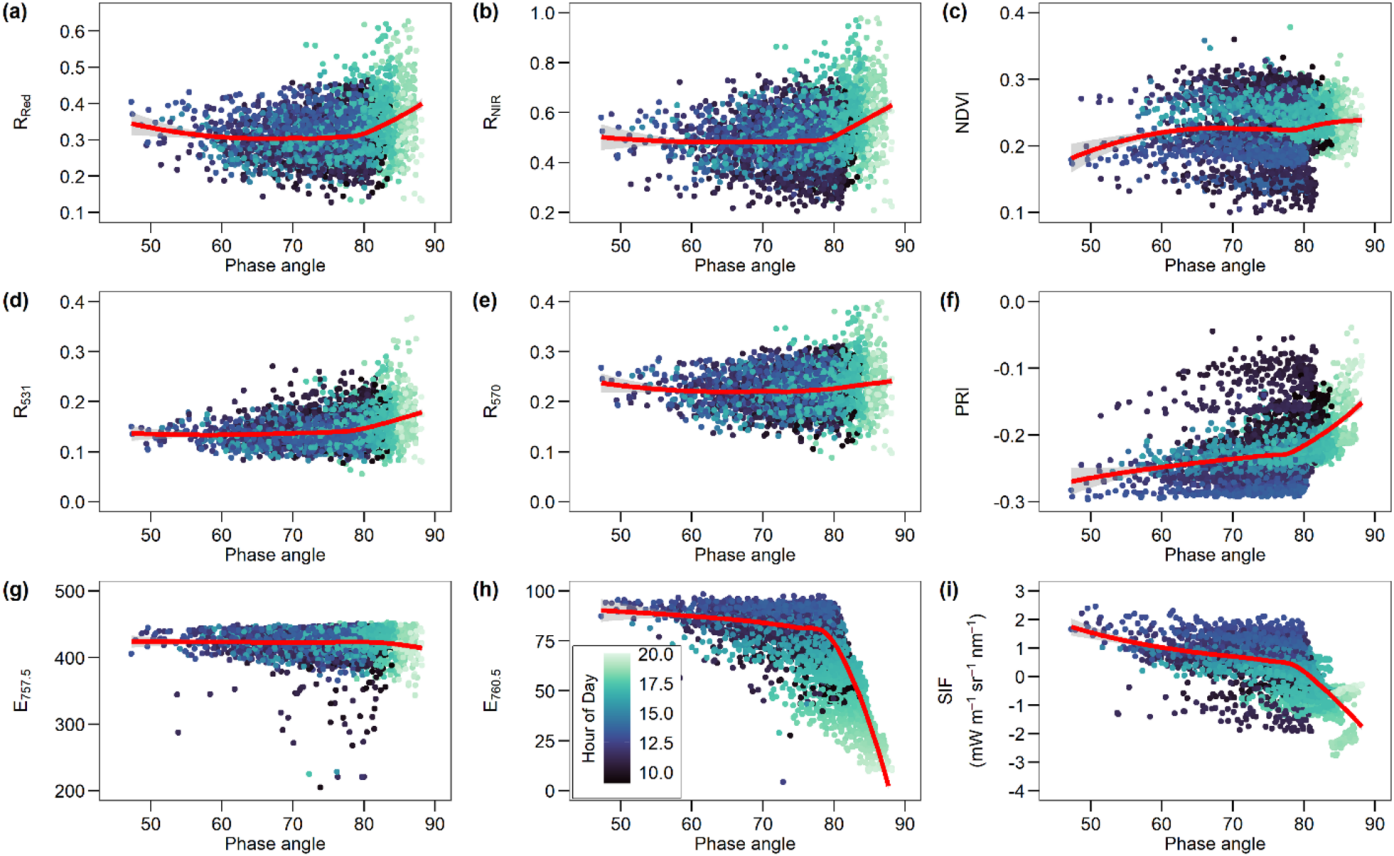
Exploring sun-sensor phase angle effects on soil targets from June 1 to June 15 for bands used for NDVI (**a**, **b**, **c**), PRI (**d**, **e**, **f**), and SIF (**g**, **h**, **i**) calculations. Color represents hour of day and red line represents LOESS fitting to highlight overall pattern

This instrument was designed for high stability and to maximize signal to noise ratio for detecting subtle variation of hyperspectral data related to canopy structure and function. However, a major consideration is the influence of solar and sensor viewing angle [78]. Depending on the viewing geometry, bidirectional reflectance distribution function (BRDF) corrections may be needed. To explore this, we determined a phase angle, which considers both solar and sensor viewing angles [43, 79], from soil targets prior to seed germination and sprouting to assume comparable reflectance targets (Fig. 7). The variation of individual wavebands and vegetation indices across phase angles was relatively stable until a phase angle of 80°, which was associated with late day (after ~ 17 h) measurements (Fig. 7). This suggests that phase angle may be useful as a data quality flag for data omission or establishing the need for angular corrections past phase angle thresholds (i.e., > 80°). Interestingly, morning measurements (before ~ 11 h) also displayed a higher scattering relative to the solar noon window (11 to 16 h). Ideally, a pure Lambertian surface would be used in the field to better understand the BRDF impact on phase angle, but this is not practical in many field locations, where a mostly dry, homogenous soil cover could be used. Our results suggest that BRDF corrections are required for diurnal tracking but much less so for seasonal tracking, which is traditionally screened to daily solar noon means. In addition to sun and sensor geometry, our system may be limited at low sun angles based on the internal diffuser setup (within the telescope enclosure), which may lead to partial illumination of the diffuser and ultimately decoupling in the sky vs target measurement. An alternative diffuser setup to avoid this is an externally fixed diffuser located outside of the telescope enclosure, however this may lead to diffuser cleanliness issues and bird perching.

Another consideration is the influence of canopy structure. PRI and SIF are highly dynamic and sensitive to sun/shade effects within a canopy [48, 80–82]. Accounting for light interception of the spectral target may help account for some of the structural effects by normalizing PRI or SIF (e.g., relative SIF) with reflected light [32]. In addition, using NDVI may be a good data quality screen for sufficient greenness and LAI as some plots did not properly germinate resulting in a delay in development and full canopy closure, potentially resulting in negative diurnal SIF values from soil contributions (Fig. 3f). This structural effect was mostly minimal in our experiment as most of our bean canopy was fully closed after maturity, but future exploration is needed and will be more necessary in other plant systems.

## Conclusions

This paper provides technical details of the mobile tower-based remote sensing system TSWIFT and highlights potential applications for collecting continuous and automated hyperspectral and SIF data for assessing spatiotemporal variation and for use in high-throughput phenotyping across a broad array of genotypic and phenotypic variation. Compared to other remote sensing tools from handheld instrumentation and drones which require operational personnel, or airborne and satellites with limited spatial (in the meters) and temporal (daily coverage) scales, our tower-based system enables automated, and high spatial (30 to 60 cm) and temporal (sub daily) resolutions. The high temporal resolution enables real time monitoring of short-term diurnal and long-term seasonal variation of vegetation. The high spatial resolution enables spot target monitoring (e.g., individual plants and/or plots) of vegetation for applications in high-throughput phenotyping or scaling with larger ecosystem scale tower (e.g., eddy covariance) and satellite footprints. The hyperspectral resolution enables a suite of vegetation indices to be calculated (e.g., NDVI and PRI), SIF retrievals, and enables plant trait prediction using full range (visible and NIR) hyperspectral data. Automated systems like this will provide optical insights for assessing structural and physiological variation of vegetation or plant breeding populations that underlines plant functional dynamics in response to local environmental conditions and genotypic resilience.

## Data Availability

The datasets used during the current study are available from the corresponding author on reasonable request.
